# Magnetic Nanoparticles Cross the Blood-Brain Barrier: When Physics Rises to a Challenge

**DOI:** 10.3390/nano5042231

**Published:** 2015-12-11

**Authors:** Maria Antònia Busquets, Alba Espargaró, Raimon Sabaté, Joan Estelrich

**Affiliations:** Department of Physical Chemistry, Faculty of Pharmacy, University of Barcelona and Institute of Nanoscience and Nanotechnology (IN2UB), Avda. Joan XXIII, 08028 Barcelona, Spain; E-Mails: mabusquetsvinas@ub.edu (M.A.B.); aespargaro@ub.edu (A.E.); rsabate@ub.edu (R.S.)

**Keywords:** magnetic nanoparticles, IONs, blood-brain barrier

## Abstract

The blood-brain barrier is a physical and physiological barrier that protects the brain from toxic substances within the bloodstream and helps maintain brain homeostasis. It also represents the main obstacle in the treatment of many diseases of the central nervous system. Among the different approaches employed to overcome this barrier, the use of nanoparticles as a tool to enhance delivery of therapeutic molecules to the brain is particularly promising. There is special interest in the use of magnetic nanoparticles, as their physical characteristics endow them with additional potentially useful properties. Following systemic administration, a magnetic field applied externally can mediate the capacity of magnetic nanoparticles to permeate the blood-brain barrier. Meanwhile, thermal energy released by magnetic nanoparticles under the influence of radiofrequency radiation can modulate blood-brain barrier integrity, increasing its permeability. In this review, we present the strategies that use magnetic nanoparticles, specifically iron oxide nanoparticles, to enhance drug delivery to the brain.

## 1. Introduction

Despite the extremely large surface area of the 100 billion capillaries contained in the human brain (approximately 20 m^2^), the capacity of many substances to pass from blood to brain is low due to the existence of the blood-brain barrier (BBB). This barrier, no equivalent of which exists in circulation through other organs, consists of tightly interconnected endothelial cells that form the circumferential interior lining of the walls of the cerebral blood vessels. The brain capillary endothelial layer is morphologically distinct from the endothelial cells in the rest of the body due to the absence of fenestrations, reduced pinocytic activity and more extensive tight junctions [1]. The tight junctions of the BBB cause a much higher transendothelial electrical resistance (TEER) than that of other tissues, which reduces intercellular space and makes it less permeable with regard to aqueous-based paracellular transport. The tight junctions are composed of smaller subunits formed by proteins [2], which, in turn, are bound to the endothelial cells by other proteins. Moreover, the BBB is equipped with many proteins, such as P-glycoprotein (P-gp) and multidrug resistance protein (MRP), that act as efflux proteins. These proteins are ATP-dependent and pump many foreign substances out of cells, including many therapeutic agents.

The BBB prevents harmful substances from entering the brain. In this way, it protects against infiltration by bacteria, viruses and other foreign material [3], and, by extension, it precludes most molecules from entering the central nervous system (CNS). According to Pardridge [3], more than 98% of small molecules, including therapeutics, do not cross the BBB. Only some gases (O_2_ and CO_2_, for example) and some small lipophilic substances diffuse freely across the BBB (passive transport). Other substances, such as glucose and amino acids, which are crucial for neural functioning, are allowed to pass into the brain extracellular fluid by selective transport, that is, by receptor-mediated transport (RMT) or carrier-mediated transport (CMT). The movement of large peptides and proteins across the BBB is facilitated by RMT. The insulin receptor, the transferrin receptor, and the insulin-like growth factor receptor are examples of endogenous receptors located at the BBB.

CMT carries small molecules (*M*_w_ < 600 Da) as it delivers nutrients, vitamins, and hormones to the CNS. A third mechanism by which substances may cross the BBB is vesicular transport, which is facilitated by either receptor-mediated or adsorptive-mediated transcytosis, possibly induced by cationic proteins [4].

Effective treatment of most CNS diseases requires the delivery of therapeutic agents to the brain. For this, a drug must cross the BBB in pharmacologically significant amounts, and this is only possible if the drug has the following characteristics: (1) a molecular mass less than 400 to 500 Da; and (2) a high lipid solubility. In particular, this is the case for psychiatric drugs. Unfortunately, many drugs that could be useful for CNS disorders cannot be given because they do not cross the BBB. Numerous attempts have been made to achieve efficient delivery of drugs to the CNS; strategies employed are invasive or noninvasive [5] An invasive method would involve overcoming the physiological barrier of the BBB through temporary osmotic opening, intracerebral infusion or intracerebral implantation (e.g., drug loaded wafer) [6,7]. Transcranial delivery can only deliver drugs near the injection site and, moreover, is ineffective if a uniform distribution of the drug to the entire brain is required [6]. All the invasive procedures are associated with high risk [3,8]. Moreover, hyperosmolar or transcranial techniques are surgical procedures that are not amenable to daily use. A noninvasive method consists of chemical modification of drugs (*i.e.*, conjugation of cell-penetrating peptides), inhibition of efflux transporters and delivery through endogenous transporters (*i.e.*, carrier-mediated transport for glucose or amino acids). The above-mentioned methods offer improvements of treatment outcomes. However, these methods are also associated with side effects. Therefore, finding ways to administer drugs effectively and safely to the CNS is a formidable challenge.

Over the last decade, attention has focused on nanoparticles (NPs) as new drug delivery agents that can transport drugs across the BBB and increase their uptake in the brain [9]. The important advantages of NP-drug complexes over their corresponding free drugs are mainly due to prolonged blood circulation. Increasing the duration of the circulation of a drug in the blood increases its capacity to interact with specific transporters or receptors expressed on the luminal side of the BBB endothelial cells and, consequently, to cross the BBB. Another important advantage of any nanotechnological approach, compared with the administration of a free drug, is that the important requirement of reaching the CNS while producing few systemic effects can be achieved by supporting parts of the nanoscale complex.

Poly(butyl cyanoacrylate) (PBCA) NPs were the first NPs that were used for *in vivo* delivery of drugs to the brain. This polymer has the advantage of being extremely rapidly biodegraded. Various modifications have been employed to increase the efficacy of these NPs across the BBB. One of the most used modifications has been overcoating the NPs with a surfactant, the polysorbate 80. The first drug that was delivered to the brain using these NPs was the hexapeptide dalargin (sequence: YAGFLR); a leucine-enkephalin analogue with opioid activity that does not cross the BBB by itself [10,11]. Other drugs that, after incorporation in polysorbate 80-coated PBCA NPs, have been transported across the BBB are the dipeptide kytorphin, loperamide, tubocurarine, doxorubicin, tacrine, and rivastigmine, among others [12,13].

Among the possible BBB penetration routes available to NPs, RMT has been shown to be the most efficient transport mechanism. RMT takes place on the luminal side, after which the compound moves through the cytoplasm of the endothelial cell and is finally exocytosed into the brain capillary endothelium. This mechanism requires the surface of the NPs to be coated with endogenous ligands or peptidomimetic antibodies. Commonly used endogenous ligands are transferrin [14,15], lactoferrin [16,17], the low-density lipoprotein (LDL) receptor [13] and insulin [18].

In the context of using NPs to improve penetration of the BBB, magnetic nanoparticles (MNPs) are of special interest, since brain cells are quite sensitive to MNPs, compared to, say, liver and heart cells [19,20]. The responsiveness of MNPs to an external magnetic field is an important factor in facilitating uptake by brain cells. Moreover, the permeability of the BBB between adjacent endothelial cells (tight junctions) is known to increase in response to physiologically relevant temperature increases (38–39 °C) [21,22]. In this way, the moderate heat dissipated from MNPs under the effect of a low radiofrequency (RF) field can increase the permeability of the BBB without perturbing other brain cells.

In this paper, we review the use of MNPs, specifically of iron oxide nanoparticles (IONs), as an appealing strategy that facilitates crossing of the BBB in therapeutic or diagnostic applications.

## 2. Magnetic Nanoparticles

MNPs are currently used in various biomedical applications: as a contrast agent for magnetic resonance imaging (MRI) [23], to induce hyperthermia in tumor therapy [24], for cell labelling and cell separation [25], in targeted therapeutics [26], in magnetofection [27], *etc*. However, uses of MNPs in the brain have been somewhat limited, due in part to a 30–50 cm working distance requirement for humans and FDA limits on the magnetic field strength used on human subjects (8 T for adults, 4 T for children) [28].

All materials present some extent of magnetism; the extent varies depending on their atomic structure and the temperature. The magnetic properties arise from the electrons circulating around atomic nuclei, from the electrons spinning on their axes and from the rotating positively charged atomic nuclei. Nuclei and electrons constitute magnetic dipoles, also called magnetons. Altogether, these effects may cancel out such that a given type of atom may not be a net magnetic dipole. If they do not fully cancel out, however, the atom is a permanent magnetic dipole. This is the case of iron atoms. The strength of a magnetic dipole is called the magnetic moment and may be thought as the quantity that determines the torque it will experience in an external magnetic field. Materials with stable magnetic properties are called ferromagnets. If the magnetic properties only exist in the presence of an external magnetic field, the material is a paramagnet. A ferromagnet becomes a paramagnet above a certain temperature called the Curie temperature (*T*_C_): the temperature at which there is a change in the direction of the intrinsic magnetic moments.

**Figure 1:**
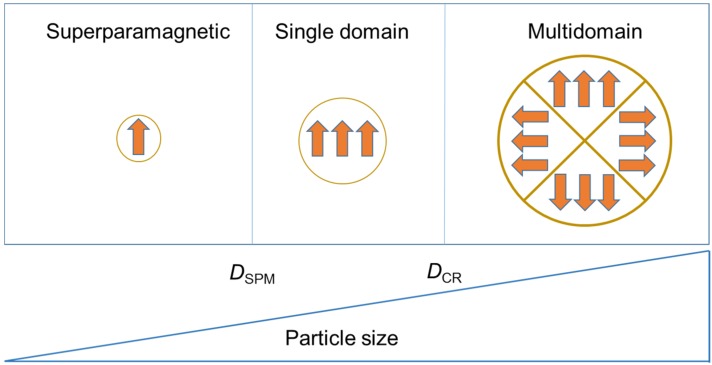
Magnetic regimes of ferromagnetic materials as a function of their size (superparamagnetic, single domain, multidomain).

Fundamental changes occur in magnetic materials when their physical size is reduced. In ferromagnetic materials, magnetons are associated within groups called domains. A magnetic domain is a volume of material in which all the magnetons remain aligned in the same direction by exchanging forces (Figure 1). The reduction of the size of a ferromagnetic material below a critical value, the so-called critical diameter *D*_CR_, becomes the material in a single domain. The value of *D*_CR_ is typically a few tens of nanometres and depends on the material. A single domain particle presents all the spins aligned in the same direction. If the size is further reduced, below the superparamagnetic diameter, *D*_SPM_, the ferromagnetic material is transformed into a superparamagnetic one. In this case, the magnetic properties are only evident in the presence of an external magnetic field. As for *D*_CR_, the value of *D*_SPM_ depends on the material.

IONs are a subtype of MNPs that are highly magnetizable and have a core of iron oxide particles composed of magnetite (Fe_3_O_4_) or maghemite (γ-Fe_2_O_3_). This core exerts a low level of toxicity as it gradually degrades to Fe^3+^ in the body and is integrated into the iron stores of the body, which are used for metabolic processes and eventually eliminated from the body.

To date, a variety of synthetic methods, such as co-precipitation, thermal decomposition, hydrothermal and solvothermal syntheses, sol–gel synthesis, microemulsion, ultrasound irradiation and biological synthesis, have been used to produce IONs. The methods of synthesis can be divided into aqueous and non-aqueous routes. Aqueous approaches are characterized by their low cost and sustainability; however, there is a generic challenge in directly obtaining water-soluble monodisperse superparamagnetic IONs (SPIONs) with no size selection. Non-aqueous routes generally yield SPIONs that only dissolve in non-polar solvents [29]. The most usual and straightforward method for synthesizing SPIONs is the co-precipitation of Fe^2+^ and Fe^3+^ aqueous salt solutions by the addition of a base. Control of the size, shape and composition of SPIONs depends on the type of salts used, the Fe^2+^ to Fe^3+^ ratio, and the pH and ionic strength of the media [30]. A major advantage of the properties of SPIONs is that they are only magnetized in the presence of a magnetic field. A magnet can therefore be used to direct the delivery of the SPIONs to a given target zone [31]. Under the influence of the magnetic field, the SPIONs are moved toward the magnet and concentrate near its location.

One of the first studies that related SPIONs with the permeability of the BBB was by Rousseau *et al.* [32]. As MNPs cannot cross the BBB, it was opened by intracarotid injection of mannitol. The study showed that SPIONs crossed the BBB 12 h after mannitol injection, at a time when brain permeability for molecules had returned to normal. Recently, Sun *et al.* [33] found that two formulations of SPIONs were unable to permeate a monolayer of bEnd.3 cells. Only after disrupting the tight junctions with d-mannitol did the flux of SPIONs across the cells increase.

**Figure 2:**
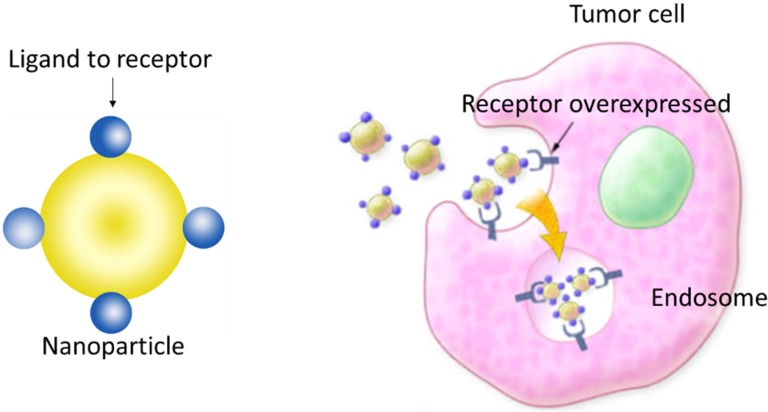
After disrupting the blood-brain barrier (BBB), nanoparticles (NPs) can accumulate at the tumor site. Receptor-mediated endocytosis of the functionalized NPs by cells overexpressing a receptor can retain NPs inside the tumor.

Owing to their inability to cross the BBB, studies with MNPs are in general based on three kinds of strategies. In the first, SPIONs are modified with functional ligands that target specific receptors in brain cells (examples of these ligands are antibodies, peptides and proteins) (Figure 2). In the second, an external magnetic field is applied to direct the movement of a SPION-encapsulated cargo to the brain [34]. Finally, the third strategy consists of applying a regulated RF field to the MNPs to produce heat and thereby transiently and locally open the BBB. Strategies that are specific to MNPs are the second and the third; both use physical principles derived from the magnetic properties of this kind of material: the magnetic force and heat generated by the particles under the influence of external electromagnetic radiation. Broadly speaking, the first strategy is no different from that adopted with other NPs to which a specific ligand is attached to the particle surface. However, this strategy can be combined with the second when an external magnet reinforces the vectorization achieved with the ligands.

The transmembrane glycoprotein NMB (GPNMB) is a protein that in humans presents two isoforms: the isoform *a*, which has 572 amino acids, and the isoform *b*, which has 560 amino acids. GMPB promotes breast tumor growth and metastasis, and its expression in tumor ephitelium correlates with poor prognosis in breast cancer patients. GPNMB is expressed in various cell types, including malanocytes, osteoclasts, osteoblasts, and dendritic cells. Moreover, GPNMB is overexpressed in various cancer types (*i.e.*, its overexpression is associated with glioma) [35]. GPNMB showed more than a 10-fold increase in the induction of protein expression compared to normal brain samples in cases of glioblastoma multiforme, the highest-grade glioma. Patients with glioblastoma multiforme have an average survival time of only around one year after diagnosis [36]. Thus, the overexpression of GPNMB is a promising target for immunotherapy for glioblastoma multiforme.

Another receptor that is also a promising marker for malignant gliomas is the epidermal growth factor receptor (EGFR). Several mutant forms of this receptor have been reported; EGFRvIII is the most common. In contrast to other EGFRs, the mutant form EGFRvIII is tumor-specific; it is not expressed in normal tissues. Hadjipanayis *et al.* [37] reported the possibility of targeting glioma cells with IONs conjugated to an antibody that selectively binds to EGFRvIII. Those authors demonstrated the accumulation of NPs inside the tumor after convection-enhanced delivery with subsequent inhibition of GBM. However, that approach has limited clinical relevance due to the local application of NPs. Furthermore, the specific antibodies only targeted a subpopulation of EGFRvIII-positive cells; they did not affect subsets of tumor cells with wild-type EGFR or other mutant receptor forms. Thus, the application of epidermal growth factor (EGF), which is one of several natural ligands for EGFR, could help cover all of the subsets of tumor cells that express wild-type EGFR, as well as its mutant forms. Several studies have showed the utility of targeting tumors in various models via the conjugation of EGF to NPs [38,39]. Shevtsov *et al.* [40] demonstrated the possibility of targeting EGFR-overexpressing brain tumors via MNPs conjugated to EGF in a C6 glioma model. In the same cellular model, Kievit *et al.* studied targeted gene delivery in a xenograft mouse model using NPs labelled with chlorotoxin (CTX) [41]. The nanovector they developed consisted of an ION core, coated with a copolymer of chitosan, PEG, and polyethylenimine (PEI). DNA-encoding green fluorescent protein (GFP) was bound to the NPs, and CTX was then attached using a short PEG linker. NPs without CTX were also prepared as a control. Mice bearing C6 xenograft tumors were injected via an intravenous (IV) route with the DNA-bound NPs. NP accumulation at the tumor site was monitored using MRI and analysed by histology, and GFP gene expression was monitored through Xenogen IVIS fluorescence imaging and confocal fluorescence microscopy. Interestingly, the CTX did not affect the accumulation of NPs at the tumor site, but it specifically enhanced their uptake into cancer cells as evidenced by higher gene expression. These results indicated that this targeted gene delivery system could potentially improve treatment outcomes of gene therapy for glioma and other deadly cancers.

As indicated above, lactoferrin can intervene in receptor-mediated transcytosis [42]. In this way, lactoferrin was covalently conjugated to PEG-coated SPIONs, and the efficacy of these conjugates at crossing the BBB was evaluated in an *in vitro* model based on primary porcine brain capillary endothelial cells (PBCECs) [43]. The results revealed that the lactoferrin-SPIONs exhibited an enhanced capacity to cross the BBB compared to the PEG-SPIONs. In good agreement with the *in vitro* results, further *in vivo* animal experiments showed a similar tendency. Other ligands that have been used to trigger receptor-mediated transcytosis are ferritin [44] and transferrin [44,45,46].

## 3. SPIONs Modified with Penetrating Peptides that Help to Penetrate Brain Cells

Cell-penetrating peptides are short peptides (generally not exceeding 30 residues) that have the capacity to ubiquitously cross cellular membranes, with very limited associated toxicity, without the need for chiral recognition by specific receptors [47].

The BBB-penetrating peptide, angiopep-2 (ANG, sequence: TFFYGGRGKRNNFKTEEY), was conjugated onto the surface of Pluronic F127-modified water-dispersible poly(acrylic acid)-bound iron oxide (PF127-PAAIO). The ANG-PF127-PAAIO complex showed a better feasibility than PF127-PAAIO in U87 cells (negligible cell cytotoxicity, better cellular uptake, and higher T_2_-weighted image enhancement) [48]. Using an *ex vivo* BBB model, those authors showed that it was more permeable to ANG-PF127-PAAIO, and therefore this complex was more able to bypass the BBB. This is because ANG-PF127-PAAIO has dual targeting capacity due to its recognition of the LDL receptor-related protein, which is overexpressed in both BBB and glioblastoma cells and the clathrin-mediated receptor on the U87 surface.

Another penetrating peptide is the trans-activator of transcription (Tat) protein derived from the human immunodeficiency virus (HIV) (sequence: YGRKKRRQRRR). The HIV-1 Tat peptide interacts with tight junction proteins and adhesion molecules. Such interaction results in an enhancement of the BBB permeability [49]. In this way, when Tat was used to functionalize magnetic liposomes (MLPs), the permeability of the BBB enhanced in comparison with non-functionalized MLPs when a magnetic field was applied. Moreover, MLPs accumulate significantly at a spinal cord injury site [50].

Recently, Ausciaux *et al.* [51] prepared ultra-small SPIONs functionalized with peptides that present an affinity for β-amyloid peptide independently of its state of aggregation. Their results have demonstrated that a SPION coupled to a cyclic heptapeptide (sequence: c-IPLPFYN-c) is capable of crossing the BBB of NMRI mice without any facilitating strategy. Around the same time, Cheng *et al.* [52] demonstrated that curcumin (Cur) can naturally bind to the surface of SPIONs, and that the particles showed low cytotoxicity and exhibited BBB penetration potential in an *in vitro* monolayer cell permeability test. *In vivo*, the particles can penetrate the BBB of both the Tg2576 Alzheimer’s disease (AD) model and non-transgenic mice. Cur-SPIONs bind to amyloid plaques in mouse brains, as shown by multiple detection methods. T_2_* *ex vivo* MRI revealed more dark spots in transgenic mice than in controls, and an important number of the detected dark spots were aligned with amyloid plaques on immunohistochemically dyed sections matched with magnetic resonance images. Techniques such as iron staining, fluorescence or immunohistochemistry showed co-localization of SPIONs and Cur on amyloid plaques. Therefore, Cur-SPIONs are novel NPs with potential use for visualizing amyloid plaques in AD patients. Further *in vivo* MRI tests in AD mice models is required to further elucidate the potential of Cur-SPIONs for early diagnosis of AD.

## 4. Transport of MNPs through Brain Cells by the Effect of an External Magnet

The magnetic force $\vec{F_{M}}$ experienced by a magnetic particle in an applied magnetic field $\vec{B}$ is given by the following equation:

(1) $$\vec{F_{M}}=\left(\vec{m}\cdot \nabla \right)\vec{B}$$

where $\vec{m}$ is the magnetic moment of the particle, calculated using the equation:

(2) $$\vec{m}=\rho V\vec{M}$$

where, in turn, ρ is the particle density, *V* is the volume of magnetic material in the particle, and $\vec{M}$ is the magnetization of the particle. According to Equation (2), the magnetic force experienced by a particle is proportional to ~*r*^3^; it increases with the size of the MNP. Therefore, high magnetization and a large size are necessary to ensure a strong attractive force. However, the size of the MNPs should remain small enough for them to be within the superparamagnetism regime.

### 4.1. In Vitro Studies

**Figure 3:**
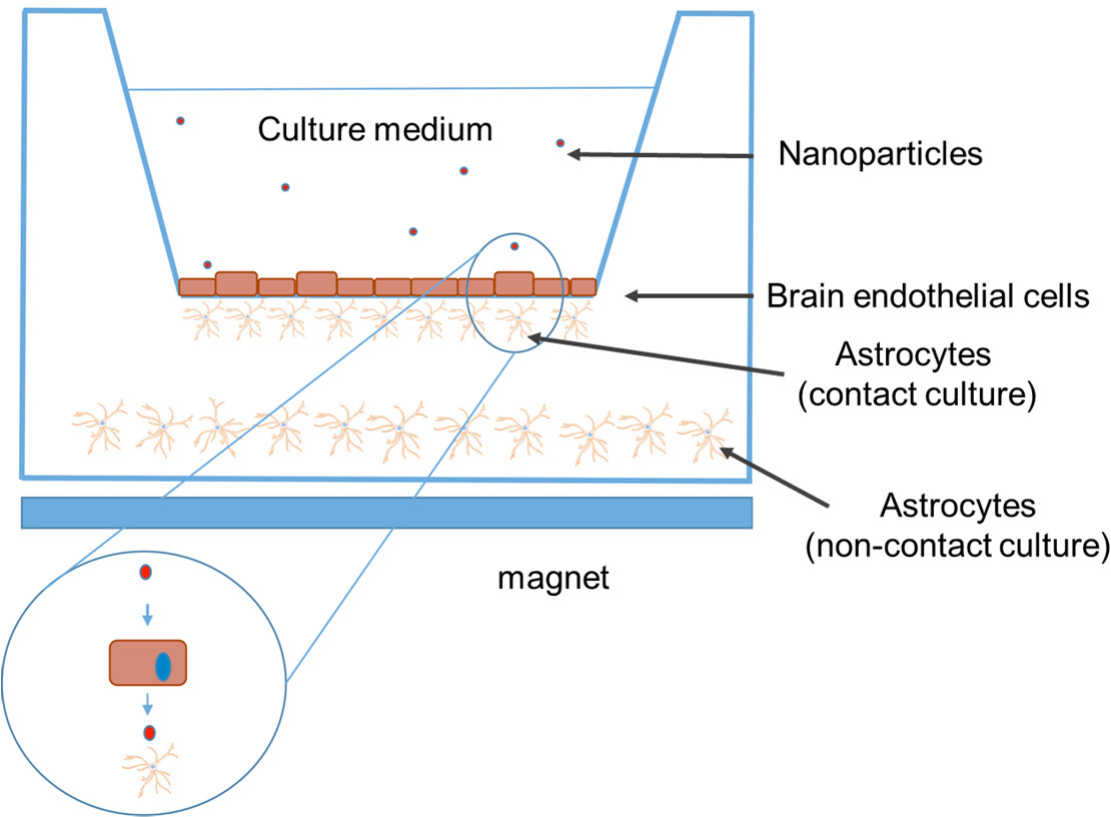
Cellular model for checking the passage of drugs or NPs across the BBB. The Transwell filter consists of a porous membrane support submerged in a culture medium. Two different Transwell co-culture modes exist: non-contact and contact culture. In non-contact culture, the cells (for instance, brain endothelial cells and astrocytes) are co-cultured in two different compartments (insert membrane in well). In contact culture, astrocytes are first seeded onto the abluminal side of the inverted Transwell filter, and after adhering, the filter is flipped back, and the astrocytes are cultured for 2 days. At the end of the second day, brain cells are seeded onto the luminal side of the Transwell filter and co-cultured with astrocytes for an additional 3–4 days. The number of NPs is determined in both compartments. Superparamagnetic IONs (SPIONs) are depicted as red dots. The passage through the BBB model can be mediated by the effect of a magnet located underneath the plate.

The capacity of any drug to pass through the BBB *in vitro* is checked in a cellular model (the BBB model). This model consists of 2-compartment wells in a culture plate with the upper compartment separated from the lower by a membrane with a pore diameter of 3 μm (Transwell™, Corning, MA, USA). On the upper side of a 24-well cell culture insert (surface area 0.3 cm^2^), endothelial cells grow to confluency, while a confluent layer of human astrocytes grow on the underside (Figure 3). The integrity of a monolayer of cells can be checked by TEER measurements of tight junctions. An average TEER value of 150 to 200 Ω/cm^2^ of cell culture insert is consistent with the formation of the BBB.

HIV-1 seems to be harboured in the brain, as pointed out by the presence of considerable quantities of unintegrated viral DNA in the brain of HIV-infected individuals [53]. The inability of antiretroviral therapy to cross the BBB provokes the prevalence of a number of CNS disorders collectively known as neuroAIDS [54]. Several studies report monocyte/macrophage-based nanocarrier drug delivery systems for delivering antiretrovirals to the brain [55,56]. This approach uses the capacity of phagocytes to cross the BBB. Likewise, Jain *et al.* reported that monocytes/neutrophils mediated delivery of Arg-Gly-Asp (RGD)-anchored MLPs to the brain [57]. Saiyed *et al.* reported that MNPs encapsulated in liposomes are captured by monocytes and moved through an *in vitro* BBB model by an external magnet without modifying the TEER values [58].

To understand the cellular uptake of MNPs (polystyrene nanospheres with trapped MNPs), Kong *et al.* [59] imaged human brain endothelial cells (D3) treated with MNPs and studied them using atomic force microscopy. This technique demonstrated that the MNPs were internalized by the cells, suggesting that trans-cellular trafficking may be the mechanism by which the MNPs cross the BBB. That study supported a model for the use of external magnets to regulate the distribution of MNPs in *in vivo* models where delivery through an intact BBB is advantageous. Moreover, the study concluded that the uptake of MNPs into the brain does not seem to need any major disruption of the endothelial barrier or modification of BBB integrity.

Thomsen *et al.* studied the capacity of fluorescent IONs to pass through human brain capillary endothelial cells facilitated by an external magnet [60]. The MNPs consisted of a magnetite core surrounded by a lipophilic fluorescent dye covered by a hydrophilic polysaccharide matrix of starch made up of α-d-glucose units. The BBB model was a monoculture of cells in inserts in 12-well Transwell membrane culture plates that contained astrocytes. A ferrite block magnet was placed underneath the culture plates. IONs passed into and through a cellular monolayer and reached the astrocytes cultured at the bottom of the chamber in a manner that was significantly enhanced by the use of an external magnet. This passage happened in a concentration-dependent manner. The magnetic force did not alter the integrity of the endothelial monolayer, nor was cell viability altered by the IONs or by the magnetic force dragging the NPs through the cells. When IONs were added to the luminal side of BBB model, they were found in astrocytes co-cultured at a distance on the abluminal side, pointing out that the particles were transported through the barrier and capturated by the astrocytes.

In another study, MLs were used as nanoparticulate systems to study the passage through the BBB. MLs with transferrin were applied to an *in vitro* BBB transmigration study in the presence or absence of an external magnetic force. Compared to magnetic force- or transferrin receptor-mediated transportation alone, the combination of the two resulted in synergistic 50%–100% increased transmigration, without affecting BBB integrity [61].

### 4.2. In Vivo Studies

MLPs show a preference for blood monocytes/neutrophils, and this results in the incorporation of magnetism into these cells, which subsequently become magnetized cells that can respond to a magnetic field [62]. In this way, Jain *et al.* showed that RGD-coated MLPs encapsulating diclofenac imparted magnetic properties to monocytes/neutrophils; and, due to their trend to migrate exclusively towards inflammatory sites, under the direction of an external magnetic field, the drug could be actively targeted to any poorly accessible site such as the brain [57].

Magnetic therapy using MNPs increased the survival of glioma-bearing rats by intensifying the brain concentration of paclitaxel [63]. Chertok *et al.* observed that intravascularly administered NPs were passively delivered to a brain tumor even in the absence of a magnetic field. A magnetic force of 0.4 T increased the concentration of starch-coated SPIONs (which accumulated approximately fivefold in a rat brain tumor than the concentration found in non-targeted (no magnetic force applied) brain tumors) [64]. Those authors observed the presence of SPIONs injected via an IV route in the brain parenchyma of normal rat brain tissue. Thus, a possible application of SPIONs for drug delivery appears to be valid, not only for BCECs, but also for neurons and glial cells located far down inside the brain, as SPIONs moving through BCECs are likely to be taken up by these cells too. This approach was improved by the administration of the NPs via a non-occluded carotid artery. This route increased the passive exposure of tumor vasculature to the NPs [65,66]. The main potential advantage of intra-arterial (IA) administration over the IV way is that the vasculature of the tissue perfused by the injected artery acquire a higher plasma concentration during the first passage through the circulation. However, IA administration in conjunction with magnetic targeting can also have a serious pitfall: The artery exposed to a magnetic field can become mechanically occluded due to NP aggregation. The extent of NP aggregation at arterial flow rates depends on the magnetic field topography and strength [67]. Therefore, the magnetic field was reduced at the carotid injection site to achieve desirable tumor retention of NPs while avoiding undesirable arterial aggregation.

**Figure 4:**
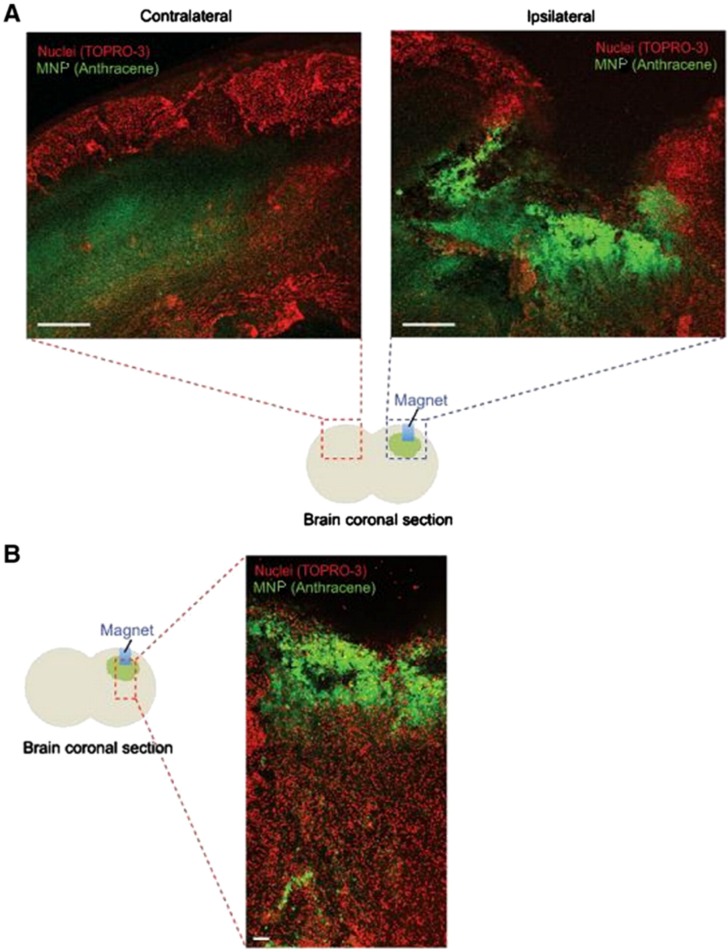
Magnetically vectored magnetic nanoparticles (MNPs) accumulate in the brain. (**A**) A small magnet was implanted in the right hemisphere of the brains of mice by stereotactic injection. (Blue represents the inserted magnet and green shade represents MNPs in cartoon). One week after implantation, MNPs were administered by IV injection. Confocal analysis demonstrated accumulation of the MNPs in the ipsilateral hemisphere, whereas a background level of MNPs was found in the contralateral hemisphere. Scale bar: 500 μm. (**B**) Confocal analysis of coronal sections of brain demonstrated enrichment of the MNPs near the magnet. Scale bar: 100 μm. Reproduced with permission of [59]. Copyright Elsevier Science, 2012.

Kong *et al.* [59] demonstrated that MNPs can cross the normal BBB when subjected to an external magnetic field. In that study, polystyrene nanospheres that encapsulate MNPs (100 nm size) and contain a fluorophore were injected into mice. In the absence of an applied magnetic field, no preferential accumulation of the MNPs within the brain was observed. To assess how MNPs respond to an external magnetic field applied locally, a Nd-Fe-B magnet was implanted in the right hemisphere of the cerebral cortex of the mice. One week post-implantation, MNPs were delivered systemically by IV injection. After obtaining brain sections, these were laser-imaged using scanning confocal microscopy to track their distribution in the brain. In the presence of the magnetic field, the number of MNPs was higher in the ipsilateral hemisphere where the magnet was implanted in comparison to the contralateral hemisphere (Figure 4A). Within the ipsilateral hemisphere, higher MNP accumulation was observed in the cortex near the magnet, whereas areas farther from the magnet exhibited lower accumulation (Figure 4B). To avoid potential tissue damage caused by the implantation of the magnet in the brain, the authors assessed whether the distribution of MNPs could be modified by the application of a non-invasive, external magnetic field (~1 T), which was applied by placing a Nd-Fe-B magnet near the head of the mice. The number of MNPs accumulated after systemic delivery was increased ~25-fold compared to control with no-magnet.

MLPs have been used to target gliomas *in vivo*. In this way, Marie *et al.* [68] subjected these NPs to selective magnetic sorting to target glioma multiforme. Such targeting was assessed *in vivo* on gliomas orthopically implanted in mice. A magnetic field gradient of 190 T m ^−1^ originated by an external 0.4 T permanent magnet located on the head of the animals effectively concentrated the MLPs in the malignant neoplasm next to the healthy brain. *In vivo* tracking of the MLPs in the brain was performed by quantitative MRI. Electron spin resonance spectroscopy was employed to calculate the iron oxide delivered to healthy parenchyma and tumor tissue *ex vivo*. Histological analysis using confocal fluorescence microscopy confirmed the significant boost in the accumulation of MLPs in the cancerous tissue up to the intracellular level. Electron transmission microscopy revealed that MLPs were effectively internalized by endothelial and glioblastoma cells. Moreover, MLPs preserved their structures.

IONs can also be encapsulated in bolaamphiphiles—amphiphilic molecules consisting of two hydrophilic headgroups linked by a hydrophobic chain. Philosof-Mazor *et al.* [69] studied the cell uptake of such vesicles in murine brain microvessel endothelial cells (bEnd.3 cells). They found that association of the SPIONs with the vesicles enhanced cell internalization, even in the absence of a magnetic field.

## 5. Increase of BBB Permeability by Magnetic Heating of Nanoparticles

MNPs present magnetic anisotropy, that is, there is a directional dependence of a material’s magnetic properties. Because of the magnetic anisotropy, the magnetic moment of a magnetic particle usually has only two stable orientations antiparallel to each other, separated by an energy barrier. The energy barrier in the simplest of cases it has uniaxial form and is given by Δ*E* = *KV*, where *K* is the anisotropy energy density and *V* is the particle volume. In superparamagnetic particles, Δ*E* is comparable to the thermal energy (*k*_B_*T*, where *k*_B_ is the Boltzmann constant and *T* the absolute temperature) and the direction of magnetization fluctuates randomly.

The application of an external alternating magnetic field (AMF) to MNPs leads to the production of energy, in the form of heat, if the magnetic field reorients the magnetic moments of the MNPs. Such an effect can be exploited to use MNPs as mediators in magnetic hyperthermia. For multidomain ferro- or ferri-magnetic materials, the production of heat is due to hysteresis losses. Since large particles of such materials contain several sub-domains, each of them has a definite magnetization direction. When the domains are exposed to a magnetic field, those domains with magnetization direction along the magnetic field axis grow, and the other ones become smaller. In single domain particles (superparamagnetic NPs) the heating is not due to hysteresis losses because such materials do not present domain walls. In this case, the external magnetic field affords energy and helps magnetic moments to rotate in overcoming the energy barrier. This energy is vanished when the particle moment relaxes to its equilibrium orientation (Néel relaxation). This fact is characterised by the Néel relaxation time *t*_N_,

(3) $$t_{\text{N}}=t_{0}e^{KV/k_{B}T\;}$$

where typical values for *t*_0_ are between 10^−9^ and 10^−10^ s.

For both types of particles, heating can also be due to the rotational Brownian motion within a liquid in which the particles are dispersed. This rotational movement creates frictional losses to the environment (Brown relaxation). Theoretically, in superparamagnetic NPs, Néel relaxation predominates over Brown relaxation, while, for larger sizes and low viscosity media, Brown relaxation is the main rotation mechanism. In the general case, both mechanisms are present, but the faster relaxation mechanism is prevailing and an effective relaxation time, τ*_R_*, can be defined as:

(4) $$\frac{1}{\tau_{R}}=\frac{1}{\tau_{B}}+\frac{1}{\tau_{N}}$$

where τ*_B_* is the Brown relaxation time.

At high AMF frequencies, the heat generated by the MNPs is enough to produce temperatures above 42 °C. At low frequencies, the heat should open up the BBB junctions. In order to make this a clinically viable technique, it is first essential to show that the opening of the BBB is local and entirely reversible.

Tabatabaei *et al.* [70,71] examined BBB permeability in rats in the presence of moderate heat dissipated via magnetic heating of MNPs by a low RF field. To verify BBB integrity, before the administration of the MNPs through the external carotid artery, fluorescent Evans blue dye was injected into the tail vein. Observation of brain samples using a 7 T magnetic resonance scanner and an epifluorescent microscope indicated a substantial but reversible opening of the BBB where hyperthermia was induced.

Dan *et al.* [72] used two types of SPIONs, cross-linked nanoassemblies loaded with IONs and conventional SPIONs, to study the effects of AMF-induced hyperthermia on SPION permeability and flux across the BBB. They used two *in vitro* modes (bEnd.3 and Madin-Darby canine kidney II cells) in Transwells™ under normothermic, conventional hyperthermic, and AMF-induced hyperthermic conditions. Their results showed that the flux across BBB models was low under normothermic condition, while AMF-induced hyperthermia for 0.5 h enhanced cross-linked-SPION cell association/uptake and flux in the absence of cell death. In contrast, SPIONs agglomerated in a cell culture medium and were taken up by, but did not flux through, the bEnd.3 BBB model. The AMF-induced hyperthermia enhanced the BBB association/uptake and permeability of cross-linked-SPIONs more effectively than conventional hyperthermia via other mechanisms in addition to the elevated temperature around the IONs. Cross-linked-SPIONs activated by an AMF produced quantifiable, controllable hyperthermia in a defined area, as required for clinical applications. In conclusion, AMF-induced hyperthermia is an approach that could potentially deliver SPIONs across the BBB with low toxicity for therapeutic and diagnostic CNS applications.

## 6. Conclusions

NPs are useful vehicles which can facilitate passage across the BBB. Three strategies can be adopted to enable the use of IONs for this purpose: modification of the IONs with functional ligands that target specific receptors of the brain cells; the use of an external magnetic field to direct the movement of the particles to the brain; and, finally, the application of an RF field to the IONs, which will generate enough thermal energy to transiently and locally open up the BBB. The last two strategies make use of two physical properties: magnetic force and the energy generated by the movement of particles. This third strategy, in particular, opens up a new avenue in the search for a local drug delivery mechanism to treat CNS disorders.
